# Editorial: Current understanding of complications associated with diabetes

**DOI:** 10.3389/fcdhc.2023.1338656

**Published:** 2023-12-06

**Authors:** Swayam Prakash Srivastava

**Affiliations:** Hartman Institute of Therapeutic Organ Regeneration, Division of Regenerative Medicine, Department of Medicine, Weill Cornell Medicine, New York, NY, United States

**Keywords:** diabetes, diabetic kidney disease, diabetic complication, diabetic endothelial dysfunction, SGLT-2 antagonists

Diabetes mellitus is a chronic metabolic disorder and is caused either by the inability of the pancreas to produce enough insulin or defects in the insulin action on the peripheral tissues, resulting in persistent hyperglycemia. Hyperglycemia is a key outcome of diabetes mellitus and chronic hyperglycemia leads to serious damage to many organs. In 2019, 48% of all mortalities caused by diabetes mellitus were observed in the subjects at or before the age of 70 years (1). The clinical data suggests that in 2015, 415 million people were supposed to develop diabetes mellitus and by 2040, this prevalence is estimated to grow to 642 million worldwide (2, 3). By the year 2050, the number of diabetic patients in the United States will increase to 48.3 million (4) although 90% to 95% of the diabetes burden is due to type II diabetes (4). Diabetes-associated complications including cardiovascular dysfunctions, renal disorders, nerve-related diseases, hepatic disorders, blindness, and organ amputation are key reasons for increased mortality among diabetic patients. Moreover, diabetes-related complexities include microvascular, macrovascular, and nerved-related issues (5). Diabetic nephropathy, retinopathy, neuropathy, cardiovascular dysfunctions, and hypertension (5) are key microvascular and macrovascular complications. Diabetic nephropathy develops in approximately 40% of diabetic patients and is the main cause of chronic kidney disease worldwide (2, 6). As a result of diabetes mellitus, kidney fibrosis develops which is the leading cause of end-stage renal disease. Diabetes mellitus-related mesenchymal activations observed in the kidneys, heart, and vessels are the critical reason for extracellular matrix (ECM) synthesis, deposition, and fibrogenesis (7). Deposition of ECM, collagens, inflammatory cells, and irregulated action of cytokines and chemokine further results in organ fibrosis in diabetes (8). There are several drug molecules known for the treatment of diabetes mellitus and related complications; however, developing safe therapeutics is still a challenge. With the development of therapeutic options for diabetes and related complications, diabetic patients have a longer life. This article gives insights into the literature on the background of the complications associated with diabetes.

Reducing the levels of oxidative stress, enhancing mitochondrial quality, and regulating the inflammatory and mesenchymal activation in diabetic-related complications have demonstrated desirable results in preclinical settings (9–11). Such medications show a new therapeutic option for the treatment of diabetes and related complications; however, further research is desirable for better management.

Researchers have reported promising activity in flavone, chalcone, and isoflavone as inhibitors against, i.e., phosphotyrosine phosphatase 1B (PTP-1B), α-glucosidase, GLP-1, dipeptidyl peptidase-4 (DPP-4), aldose reductase, and sodium-glucose co-transporter-2 (SGLT-2) in type II diabetes (11). These effective molecules have promising antidiabetic activity in preclinical settings and mouse models of diabetes. Recently, tissue-specific angiopoietin-like protein 4 (ANGPTL4), macrophage-stimulating protein 1 (MST-1) inhibitors, and SGLT-2 inhibitors have been found to have tremendous activity in controlling diabetes and related complications. Recently, we have observed that enzyme catechol-o-methyl transferase (COMT) deficiency is involved in metabolic disorders and pre-eclampsia-associated phenotypes in mice (12). COMT by-product 2-methoxy estradiol (2-ME) treatment suppressed the phenotype of metabolic disorders and pre-eclampsia in the mouse models; however, a randomized clinical trial is needed in diabetic patients for the development of 2-ME into the usable form as a medicine (12).

Endothelial cell dysfunction is a critical feature of diabetes-related complications (13). Endothelial-to-mesenchymal transition (EndMT) is the process through which endothelial cells lose the endothelial characteristics and gain the features of mesenchymal cells (14). Conversion of endothelial cells into the EndMT phenotype is the crucial process to accelerate fibrosis-associated pathways resulting in the deposition of ECM and fibrogenesis-associated proteins (15, 16). Our research group identified three key endogenous molecules that are associated to cellular stability and regulation of mesenchymal cells and fibrogenesis, and these are 1) endothelial glucocorticoid receptors (GR), 2) endothelial fibroblast growth factor receptor 1 (FGFR1), and 3) endothelial sirtuin 3 (SIRT3). Deficiency of either GR, FGFR1, or SIRT3 protein levels causes significantly diminished levels of fatty acid oxidation and activation of the inflammatory cascade in the endothelial cells itself and also to tubules in diabetic conditions, resulting in severe fibrotic responses in the diabetic kidneys and heart (17–22). Defective fatty acid metabolism is critical in renal fibrosis (23). GR regulates canonical Wnt signaling and IL-6 levels. FGFR1 signaling is important for restoring antifibrotic microRNAs such as miR-29s and miR-let-7s, whereas SIRT3 regulates metabolic flux by targeting pyruvate kinase M2 tetramer-to-dimer formation (10, 24). The cumulative effects lead to cellular leakage, defects in the permeability, EndMT activations, and related fibrosis in the kidneys and heart in the diabetic condition (24, 25). Podocyte GR plays a critical role in regulating fibrosis in the diabetic glomeruli. Podocyte GR mitigates the Wnt signaling and elevates the levels of fatty acid oxidation and, therefore, is associated with the reduction in the levels of EndMT in glomerular endothelial cells, suggesting that GR loss in podocytes disrupts the important crosstalk between podocytes and endothelial cells in diabetes (17).

In this Research Topic, Huang et al. demonstrated the crucial role of angiotensin-converting enzyme (ACE) in diabetic nephropathy and retinopathies. The authors demonstrated that elevated levels of serum ACE are related to DN progression or retina impairment in diabetic patients. This study will facilitate the identification of biomarkers for the management of diabetes-related complications. In another study, the authors discussed how hand manifestations reflect a systemic profibrotic state and are key clinical biomarkers of current or future internal organ fibrosis (Phatak et al.). A study by Sacareau et al. demonstrated the screening of CKD patients among diabetic subjects in French Guiana. This study links diabetes and hypertension, the two most obvious risk factors for developing CKD, in human subjects.

## Conclusion

This Research Topic highlights new cellular mechanisms in diabetes-related complications and identifies new molecules for the treatment of organ fibrosis, neuropathies, and foot ulcers. We speculate that this Research Topic will provide valuable information that could be used in the management of diabetes-related complications.

## Author contributions

SS: Conceptualization, Investigation, Software, Supervision, Validation, Writing – original draft, Writing – review & editing.
